# Bacterial Therapy of Cancer: Promises, Limitations, and Insights for Future Directions

**DOI:** 10.3389/fmicb.2018.00016

**Published:** 2018-01-23

**Authors:** M. Gabriela Kramer, Martín Masner, Fernando A. Ferreira, Robert M. Hoffman

**Affiliations:** ^1^Department of Biotechnology, Instituto de Higiene, Facultad de Medicina, Universidad de la República, Montevideo, Uruguay; ^2^Laboratory of Carbohydrates and Glycoconjugates, Department of Organic Chemistry, Facultad de Química, Universidad de la República, Montevideo, Uruguay; ^3^AntiCancer, Inc., San Diego, CA, United States; ^4^Department of Surgery, University of California, San Diego, San Diego, CA, United States

**Keywords:** bacterial-based therapies, Coley’s toxins, antitumor effect, immune response, bactofection, combined therapies, *Salmonella enterica* serovar Typhimurium (*S.* Typhimurium), animal models of cancer

## Abstract

Spontaneous tumors regression has been associated with microbial infection for 100s of years and inspired the use of bacteria for anticancer therapy. Dr. William B. Coley (1862–1936), a bone- sarcoma surgeon, was a pioneer in treating his patients with both live bacterial-based and mixture of heat-killed bacteria known as “Coley’s toxins.” Unfortunately, Coley was forced to stop his work which interrupted this field for about half a century. Currently, several species of bacteria are being developed against cancer. The bacterial species, their genetic background and their infectious behavior within the tumor microenvironment are thought to be relevant factors in determining their anti-tumor effectiveness *in vivo*. In this perspective article we will update the most promising results achieved using bacterial therapy (alone or combined with other strategies) in clinically-relevant animal models of cancer and critically discuss the impact of the bacterial variants, route of administration and mechanisms of bacteria-cancer-cell interaction. We will also discuss strategies to apply this information using modern mouse models, molecular biology, genetics and imaging for future bacterial therapy of cancer patients.

## Back to the Controversial Future

The use of microorganisms, in particular live bacteria, for prophylactic vaccination and cancer therapy have been used in humans for long periods in the past and have been a matter of controversy (Payette and Davis, 2001; Hoption Cann et al., 2003). Dr. William B. Coley in the 19th century at the New York Hospital, later to become the Memorial Sloan Kettering Cancer Center (McCarthy, 2006; Hoffman, 2016a), observed and reported spontaneous tumor regression in patients with streptococcal infections (principally erysipelas, known to be caused by *Streptococcus pyogenes*). In 1891 Dr. Coley started to treat his cancer patients with streptococcal living cultures and observed that inducing a fever was crucial for tumor regression; however such a strategy also caused some fatalities (McCarthy, 2006). Coley then generated a variety of “anti-tumor vaccines” mixing heat-killed bacteria, combining *S. pyogenes* with *Serratia marcescens*. In this way he could stimulate the symptoms of an infection (for example, inflammation, chills, fever) without the risks of a bacteremia. These vaccines became known as “Coley’s toxins” and were administered to patients with sarcomas, carcinomas, lymphomas, melanomas, and mielomas. Despite the cures and remarkable improvements obtained in patients treated with Coley’s bacterial-based therapeutics (Nauts et al., 1946; Nauts, 2004; McCarthy, 2006), his boss, the renowned pathologist James Ewing, forced Dr. Coley’s to end all projects involving bacteria-based treatments alleging Coley’s inconsistent data and pronouncing himself in favor of radiotherapy, which rapidly took over the market of cancer therapeutics.

Sixteen different preparations of “Coley’s toxins” have been used since the method was introduced in 1892, of which three were considerably more potent than the rest (particularly the Buxton’s Type VI formula). However, the only preparation available in the United States since 1921 seemed to be weaker compared to the used in the early years (Nauts et al., 1946). Coley’s work gradually fell out of favor and by 1962 the Food and Drug Administration (FDA) refused to acknowledge “Coley’s toxins” as an approved drug, making it illegal to prescribe them outside of clinical trials. Since then, several small clinical trials have been conducted with mixed results.

To date, Bacillus Calmette-Guerin (BCG), is the only bacterial agent approved by the FDA and it is employed for the treatment of superficial, non-muscle invasive bladder cancer (NMIBC) since the late 1970s (Gontero et al., 2010). BCG is an attenuated strain of *Mycobacterium bovis* obtained at the Pasteur Institute in the early 1900s. Patients typically receive repeated instillations of live bacteria into the bladder. BCG is recommended as the standard of care for high-risk NMIBC and remains the most effective intravesical treatment for this disease, although the response predictor factors of BCG are unknown (Kim and Steinberg, 2001; Zlotta et al., 2009; Gontero et al., 2010).

In the last decades a resurgence of the field has taken place and contemporary investigators demonstrated the efficacy of a number live attenuated bacteria to destroy cancer cells *in vitro*, to selectively accumulate, replicate within and destroy tumors in rodents, to induce an immune-mediated anti-tumor response and to target small metastatic nodules spread in the organism and inhibit their growth (Yu et al., 2004; Adkins et al., 2012; Hoffman, 2012b). Promising results were obtained using modern methods of bacterial genetics, cancer cell and molecular biology, and *in vivo* imaging (Min et al., 2008a,b; Uchugonova et al., 2012; Hoffman, 2015). The mechanism of action of bacterial therapy of cancer and toxicity *in vivo* is not yet clearly understood and the potential acquisition of antibiotic-resistance or mutations that would revert the bacteria attenuated phenotype could be a real risk for the patients. Therefore, the building of a broad integrated picture requires a critical scientific and medical vision, for moving forward.

## Highlights But Still Many Questions

Bacteria display a number of different characteristics that could be relevant in the therapy against cancer. The direct and immune-mediated anticancer properties derive from biological interactions between the bacteria and the host tumor microenvironment. Important features of the bacteria such as motility, tumor chemotaxis, invasive capacity, cytotoxic potential, pathogen-associated molecular patterns (PAMP) composition/abundance, among others, vary between strains and may affect how they trigger the anti-tumor response (Dang et al., 2001; Cheadle and Jackson, 2002; Hoffman, 2011; Adkins et al., 2012; Kim et al., 2015; Phan et al., 2015). Although the mechanism of bacterial tumor tropism is poorly understood there is evidence indicating that irregular organization of blood vessels within the tumor tissue that often leads to the development of hypoxic and/or necrotic regions and/or an immune-suppressive microenvironment inside the tumor mass may facilitate survival and growth of attenuated auxotrophic bacteria by providing them with nutrients and immune-protection (Forbes et al., 2003; Wouters et al., 2003; Yu et al., 2004). Moreover, niche-specific genes involved in the process of preferential tumor colonization after systemic bacteria delivery, were also identified (Silva-Valenzuela et al., 2014).

Different variants from the genera *Bifidobacterium, Clostridium, Lactococcus, Shigella, Vibrio, Listeria, Escherichia*, and *Salmonella* have been assayed in animal models of cancer (Yazawa et al., 2000; Cheadle and Jackson, 2002; Oelschlaeger, 2010; Patyar et al., 2010; Hoffman, 2012b). Obligate anaerobes such as *Bifidobacterium longum* and a *Clostridium novyi* strain devoid of its lethal toxin (*C. novyi*-NT) have shown preferential localization in low oxygenated necrotic areas of implanted tumors in mice after systemic administration, inducing tumor regression in some cases, although they were unable to grow in viable tumor tissue due to high oxygen tension, a fact that may have limited their efficacy as mono-therapy (Dang et al., 2001; Hoffman, 2012a). However, intra-tumor (i.t.) administration of *C. novyi*-NT has shown objective responses in canine tumors, which are more like those of humans because they are naturally occurring in animals with heterogeneous genetic backgrounds (Roberts et al., 2014). On the other hand, attenuated auxotrophic mutants of the facultative anaerobe *Salmonella enterica* serovar Typhimurium (*S.* Typhimurium) have been shown to invade and destroy a broad number of cancer cell types *in vitro*, as well as to replicate in hypoxic and oxic tumor regions *in vivo*, being the most efficient anti-tumor bacteria assayed in experimental models of cancer thus far (Pawelek et al., 1997; Leschner and Weiss, 2010; Nguyen et al., 2010; Hoffman, 2011, 2016b,c). Among them, *S*. Typhimurium VNP20009, attenuated by the lipid A (*msbB*) deletion and purine (*purI*) auxotrophic mutations, has shown anti-tumor efficacy in mice and swine and was safely administrated to patients with metastatic melanoma and renal carcinoma in a Phase I clinical trial; however, efficacy was not observed, perhaps due to over-attenuation (Toso et al., 2002).

A more tumor-virulent variant and less toxic against the host is *S*. Typhimurium A1-R (Zhao et al., 2006). Unlike VNP20009, the A1-R variant was obtained by successive passages from re-infected human tumor xenografts in nude mice treated with the *S*. Typhimurium A-1 auxotrophic (Leu- Arg-dependent) parental bacteria (Zhao et al., 2005). This selection procedure may account for A1-R’s particular tumor-specificity and stronger anti-tumor activity (Zhao et al., 2006). A comparative study between VNP20009 and A1-R in nude mice showed that mice tolerated *S.* Typhimurium A1-R to at a least twofold higher dose than VNP20009 when the bacteria were administered intravenously (i.v.). In addition, A1-R showed higher tumor targeting and inhibited the Lewis lung carcinoma to a greater extent than VNP20009, with less body weight loss (Zhang et al., 2015). In addition, *S*. Typhimurium A1-R mono-therapy has shown to be effective against primary and metastatic human prostate, breast, and pancreatic cancer as well as osteosarcoma, fibrosarcoma, and glioma in clinically-relevant mouse models (Hoffman, 2016c and references therein). Tumors with a high degree of vascularity were more sensitive to A1-R and vascular destruction appears to play a role in A1-R anti-tumor efficacy (Liu et al., 2010). In addition, A1-R was shown to induce stem-like and non-stem cancer-cell death *in vivo*, indicating that A1-R could be used to kill chemo-resistant cancer stem-like cells (Hiroshima et al., 2013). Together these results suggest that *S.* Typhimurium A1-R may have a greater clinical potential than VNP20009 (Zhang et al., 2015) and that not only the bacterial species, but also their genetic background needs to be taken into account when searching for improvements in bacteria-based therapies.

*Salmonella* Typhimurium defective in the synthesis of ppGpp (ΔppGpp: depletion of *relA* and *spoT*), showed 10^5^ to 10^6^-fold attenuation compared with WT strain (Na et al., 2006). This attenuated strain showed very high tumor targeting and stimulation of regional tumor immunity (Kim et al., 2015; Phan et al., 2015; Zheng et al., 2017).

In this regard, high-throughput screenings for *Salmonella* avirulent mutants can identify variants with reduced fitness in normal tissues but unchanged fitness in tumors for potential use as cancer therapeutics (Arrach et al., 2010). As an example, a reported genetically-engineered *S*. Typhimurium *aroA aroD* double mutant harboring the Flt3 Ligand, used to treat melanoma in mice resulted in 50% tumor regression (Yoon et al., 2007). However, *aroA* and *aroD* were later identified by Arrach et al. (2010) as Class 2 mutants which show reduced fitness in tumors compared to Class 1 mutants, increasing the probability that a different avirulent mutant that grows better in tumors might have resulted in a more complete anti-tumor response. In a competitive fitness assay in human prostate tumors growing in mice, Class 1 mutant *STM3120* not only had a fitness advantage over Class 2 mutants, but also effectively targeted tumors after intragastric delivery, suggesting an oral route as an option for bacterial cancer therapy (Arrach et al., 2010). The ability to screen thousands of candidates and evaluate individual mutants in parallel using high-throughput sequencing offers a clear advantage over conventional screening methods. Mutants that retain tumor-targeting while being poor colonizers of normal tissue, are desirable for cancer therapeutics.

The patient-derived xenograft (PDX) mouse models of cancer are emerging as an important component of personalized cancer therapy (Cho et al., 2016). PDX models are generated by implanting sectioned patient tumor fragments into immunodeficient mice, subcutaneously or orthotopically (into the organ or tissue of the cancer origin). Patient-derived orthotopic xenografts (PDOX) have the additional advantage that they usually metastasize as in the patient (Hiroshima et al., 2016). These models retain the histologic characteristics, heterogeneity of cancer cells and genomic signature of the patient tumor enabling the identification of effective individualized therapy (Cho et al., 2016). *S.* Typhimurium A1-R has shown to be effective against osteosarcoma in a PDX model (Murakami et al., 2017) and soft-tissue sarcoma, pancreatic cancer and melanoma in PDOX models (Hiroshima et al., 2014; Murakami et al., 2016; Yamamoto et al., 2016). Although these models need to be immunocompromised in order to allow human tumor engraftments and therefore do not allow evaluation of the immune-mediated bacterial activity, we believe that studies that employ PDOX models would allow the selection of the best-suited bacteria for individual tumors and prediction for its effectiveness in patients. “Humanized” PDOX models (Zitvogel et al., 2016) will be used to determine tumor-immunology effects of bacteria.

**FIGURE 1:**
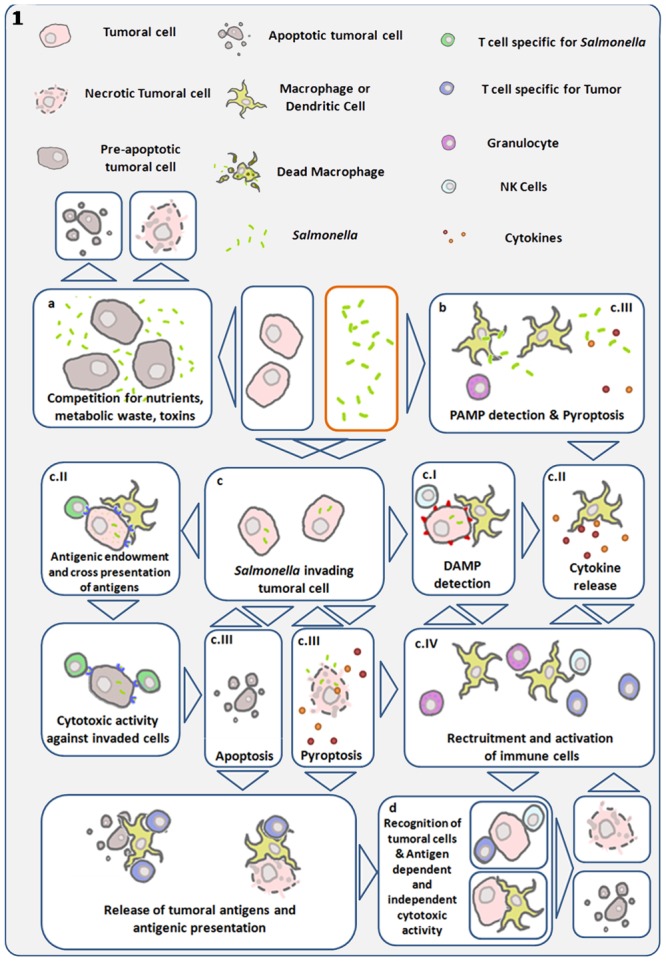
Diagram showing main antitumor mechanisms induced by *S.* Typhimurium (*Salmonella*). Links are established between direct cytotoxicity induced by bacteria and indirect tumor cell death triggered by the immune system. (a) Bacterial infection within the tumor microenvironment results in inhibition of tumor growth and cell death. (b) Detection of bacterial pathogen-associated molecular patterns (PAMP) by immune cells, trigger cytokine release and recruitment of leukocytes capable of initiating anti-tumor immune responses (Patyar et al., 2010). (c) Using their Type III secretion system, *S.* Typhimurium can introduce bacterial factors in cancer cells allowing its internalization and intra-cellular replication (Avogadri et al., 2005; Knodler et al., 2010). (cI) Invasive *Salmonella* induces cell stress responses through danger-associated molecular patterns (DAMP), which are interpreted as damage signals by the immune system. (cII) Simultaneously, this same process can lead to cytokine expression and the transfer of antigens from the bacteria to the cancer cell, enabling the adaptive immune system to recognize and target the invaded cancer cell as infected and bearer of exogenous antigens (Avogadri et al., 2005). Gap junctions are concomitantly induced in the invaded cell and enable cross presentation of antigens to antigen presenting cells (Saccheri et al., 2010). Both processes can give rise to antigen-dependent elimination of infected cancer cells. (cIII) *Salmonella* can lead to the death of the infected cell, by inducing apoptosis or pyroptosis. The later is a programmed inflammatory cell death, characterized by activation of caspase 1, activation of the inflamosome, and IL-1B and IL-18 secretion, as well as cell rounding and detachment, cytoskeleton reorganization, nucleus deformation and rupture of the cell membrane, resulting in the release of inflammatory signals (Fink and Cookson, 2005, 2007; Knodler et al., 2010; Wang et al., 2013). This mechanism can result in cancer-cell death and immune-cell activation. Pyroptosis was first described in macrophages, which die quickly as a result of this process, and is of particular interest in cancer immunotherapy, as tumor-associated macrophages have been shown to have immune-suppressive proprieties. Reducing their number could be another component of the *S.* Typhimurium anti-tumor effect. Cancer cell death leads to tumor-antigen liberation, and the released bacteria can infect surrounding cancer cells. (cIV) In the process of pyroptosis, pro-inflammatory cytokines IL1-B and IL-18 can trigger recruitment and activation of immune cells (Knodler et al., 2010; Zhao et al., 2012; Wang et al., 2013). (d) Various mechanisms enhance and converge to enable tumor-antigen recognition and activation of cytotoxic responses both in an antigen-dependent and -independent manner. *S.* Typhimurium proteins injected into the cancer cell cytosol are subject to proteasomal degradation, resulting in bacterial peptides that can be presented through MHC I to cytotoxic lymphocytes (Avogadri et al., 2005; Saccheri et al., 2010).

**FIGURE 2:**
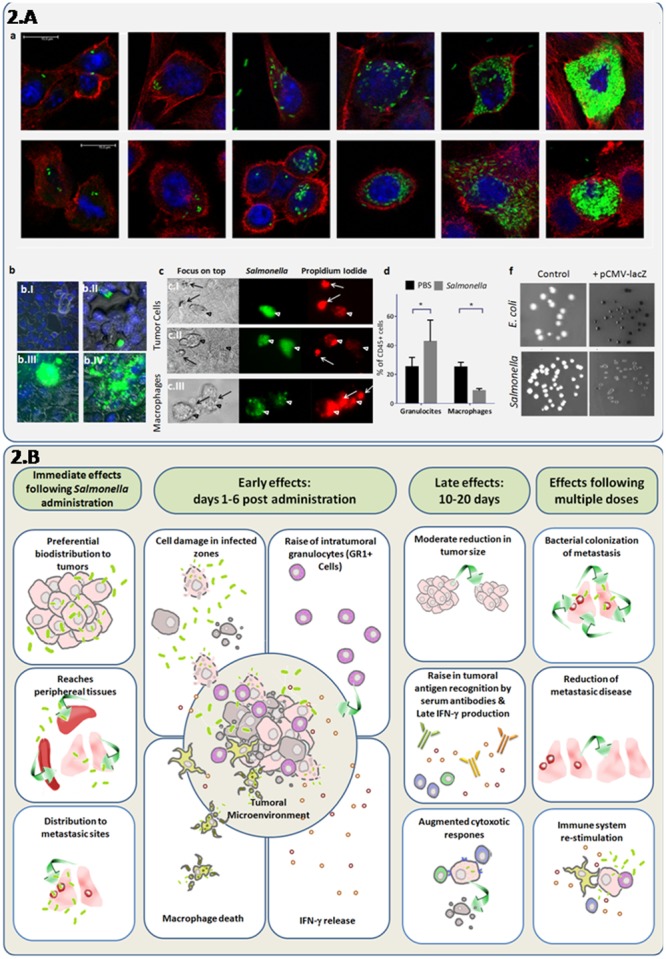
Direct and synergystic anti-tumor effects of attenuated *S.* Typhimurium integrating cellular and systemic immune responses. **(A)** Induction of cell death and granulocyte recruitment associated with intracellular replication of attenuated *S.* Typhimurium LVR01 (*Salmonella*), which previously showed a modest antitumor effect in the 4T1 metastatic breast cancer model (Kramer et al., 2015). (a) Confocal microscopy indicates bacteria invasion and replication in breast cancer cell lines: 4T1 (ATCC-CRL2539) (upper line) and NMU (ATCC-CRL1743) (lower line) in a time-course experiment. Cell cultures were grown in glass coverslips, infected with *Salmonella* expressing the GFP gene and sampled at 2, 12, 24, or 48 h to follow progression of intracellular replication. Specimens were fixed in paraformaldehyde 4%, washed in PBS and stained with DAPI and Phalloidin-Alexa555 (Invitrogen^TM^). After the staining, the coverslips were washed with PBS, mounted using Pro Long Gold (Invitrogen^TM^) and sealed with nail polish. This three color fluorescence pattern allowed the 3D analysis of the infected cultures, by simultaneously visualizing the bacteria, the nucleus and the F-actin cytoskeleton. Intracellular/extracellular determination of bacteria was possible due to the delimited borders of the actin cytoskeleton which are close to the cell membrane. Images were obtained with a LEICA^®^TCS SP5 II spectral confocal microscope and processed with the software Leica^®^LAS AF. As observed, bacterial invasion progresses, showing intracellular cytoplasmic hyperreplication over time. (b) Epifluorescence microscopy of 4T1 monolayers infected cells. Cancer cells were infected with *Salmonella*-GFP for 2 h and observed at different time points. Specimens were washed in PBS, fixed in paraformaldehyde 4%, and stained with DAPI (Invitrogen^TM^). After 5 min staining, invaded cultures were washed and observed in a Nikon^®^Ti-S epifluorescence inverted microscope. At 2 h few peri-nuclear bacteria could be seen (b.I) At 24 h (b.II) bacteria replicated in the cytoplasm and some infected cells appear rounded and extruded. At 48 h (b.III) densely-infected cells were similar, and eventually burst and release their cellular contents (b.IV). (c) Live infected cultures were observed either intact or in the presence of propidium iodide (500 nM) to assess intracellular bacterial mobility and cell viability, respectively. Monolayers of mammary cancer cells: 4T1 (c.I) and NMU (c.II), as well as macrophage cells J774.A (c.III) were infected with *Salmonella*-GFP. At 24 h post-infection, infected cells (green) die as indicated by propidium iodide staining (red). Macrophages died at earlier time points (2–16 h). Arrows point the extruded cells. (d) Flow cytometry of intratumor immune cells at 6 days after *Salmonella* inoculation of 4T1 tumors *in vivo*. As observed, the intra-tumor granulocyte/myeloid-derived-suppressor cell (Ly6G+CD11b+) levels increase and macrophage (F4/80+CD11b+) levels decreased after bacteria administration among total leukocytes (CD45+ cells). (f) X-gal agar plates were used to seed the untransformed bacteria (control) or bacteria transformed with a plasmid containing the *lacZ* gene under the control of the eukaryotic cytomegalovirus (CMV) promoter (pCMV-lacZ). As observed, the *lacZ* gene product β-galactosidase was detected, indicating that the CMV promoter was active in prokaryotic cell species. **(B)** *In vivo* effects of attenuated *S.* Typhimurium (*Salmonella*) in mice bearing metastatic cancer. This integrative diagram shows the anti-tumor effects of attenuated variants of *Salmonella* evaluated as mono-therapy. The bacteria inoculation by different routes (systemic or intratumoral) results in its biodistribution to most organs, but with a marked preference for tumors, including metastasic sites (Pawelek et al., 1997; Low et al., 1999; Forbes et al., 2003; Yu et al., 2004; Hoffman, 2016a). In tumors, bacterial infection is associated with tumor-tissue architecture deterioration, a rise in granulocytic cells and INF-γ induction and a decrease of intra-tumor macrophages (Avogadri et al., 2005; Westphal et al., 2008; Zheng et al., 2017). Late effects (10–20 days after bacteria administration) are characterized by a moderate decrease in tumor size, adaptive immune responses including INF-γ production, antibody recognition of tumor antigens, and cytotoxic immune activities (Avogadri et al., 2005; Kramer et al., 2015; Masner et al., unpublished results). Repeated administration of attenuated bacteria could result in a better targeting of metastases (Zhao et al., 2012), while stimulating immune responses that enhance cancer-cell elimination.

**Figures 1, 2** show some of the complex net of events that are involved in promoting bacterial anti-tumor efficacy. However, in most models bacteria mono-therapies are not sufficient to eliminate a primary tumor or the metastatic burden. Combined therapies including chemotherapy (Dang et al., 2001; Yamamoto et al., 2016; Yano et al., 2016), radiotherapy (Jiang et al., 2010), traditional herbal medicine (Zhang et al., 2013), anti-angiogenic and/or immunotherapy (Binder et al., 2013; Kramer et al., 2015) or the use of bacteria carrying plasmids coding for anti-tumor genes (reviewed in Moreno et al., 2010; Nguyen and Min, 2017) have shown enhanced results. Based on the use of eukaryotic gene-expression systems it has been suggested that bacteria can act as vector systems for plasmid transfer to mammalian cancer cells, a process known as “bactofection” (Weiss and Chakraborty, 2001; Baban et al., 2010; Othman et al., 2013). However, this trans-kingdom gene delivery assumption is still a matter of controversy (Gahan et al., 2009 and **Figure 2A**). Therefore, for the best performance of a bacteria + plasmid combination, the determination of the actual location of transgene expression would allow the right selection of the gene, promoter, and secretion system (if required) to achieve optimized therapy (Forbes, 2010; Zheng et al., 2017). In addition, since the bacteria usually induce death of infected cells within few hours, the rational to use bacteria as a gene delivery system (vector) to immune and/or tumor cells needs to be re-evaluated if medium- or long- term persistence of therapeutic gene expression is necessary *in vivo*.

In terms of combined therapies, a remarkable example of a neoadjuvant (pre-operatory) synergistic efficacy was observed using *S.* Typhimurium *aro C* mutant LVR01 in combination with interleukin 12 (IL-12) expressed from the alfaviral eukaryotic gene vector SFV-IL-12 (Kramer et al., 2015). This approach was evaluated in an immunocompetent mouse model of locally-advanced breast cancer and resulted in a highly effective anti-metastasic therapy, leading to 90% disease free mice, while either mono-therapy was not effective. Moreover, the efficacy of this combined therapy depended on the order in which both agents were administered (Kramer et al., 2015). An initial anti-angiogenic effect associated with a T helper-cell-1-primed response that was timely induced seemed to account for the main global effect. However, the underlying mechanisms of this combination and timing of both factors raised various questions that remain un-answered.

Other relevant questions to be answered for bacteria-based cancer therapy optimization are related to the dose, schedule, and route of administration. A dose-dependent effect of attenuated *S.* Typhimurium was observed, as well as multiple dosing are more efficient than mono-doses (Hayashi et al., 2009; Nagakura et al., 2009; Grille et al., 2014), although the range needs to be determine to avoid toxicity (Zhao et al., 2012). The efficacy and safety of three different routes of *S*. Typhimurium A1-R administration: oral (p.o.), i.v. and i.t. was compared in nude mice with orthotopic human breast cancer indicated that the p.o. route was safer, and the i.v. route was more effective (Zhang et al., 2012). However, such experiments may need to be performed for each type of tumor, since it was also shown in a model of disseminated human ovarian cancer treated with i.v. and intraperitoneal (i.p.) *S*. Typhimurium A1-R, that i.p. treatment was less toxic than i.v. administration (Matsumoto et al., 2015).

Although useful in many approaches, human xenografted tumors into immunodeficient mice limit our knowledge about the range of effects that certain bacterial strains can exert. In this regard, studies in immunocompentent animals are more representative of the complex spectrum of interactions between the bacteria and the tumor microenvironment, thereby enabling immune effects that are otherwise absent in immunocompromised mice. This could be crucial for “tunning” the bacteria to the right degree of immunogenicity/attenuation, avoiding shock while promoting adjuvant effects (Yu et al., 2004). Moreover, toxicity issues regarding immunotherapies are a main concern today. From acute shock to autoimmune diseases, we could gain a better understanding of the risk of side effects of bacteria therapy of cancer from preclinical models that include all the functional branches of the immune system. Undesirable attenuated bacterial infection can be in theory treated with antibiotics; however, long-term clinical trials in humans are required to evaluate toxicity in detail, since the chance of septic shock and/or tumor lysis syndrome could be a fact. In addition, we believe that, there is still a considerable need of work to evaluate bacteria for natural acquisition of antibiotic-resistant genes and/or reversion of attenuation mutations, as well as comparing the anti-tumor efficacy and secondary effects of bacteria or bacterial products *versus* conventional therapies. Moreover, we cannot rule out the possible clearance of bacteria by the immune system before reaching the tumor site in a patient-dependant manner, resulting in treatment failure.

## The Future of What Dr. Coley Began

Each of the 16 “Coley’s toxins” that have been used might have a complex and variable composition, including components of the culture media, products released by the bacteria in the medium, components relevant by bacteria lysis (and autolysis). The inactivation method used to prepare the vaccine and the inclusion, or not, of a filtration step in the preparation of the toxins will affect the final products. The i.v. administration of a suspension of inactivated bacteria cells may mimic a nanodrug, and the number of particles, their size, shape, charge, and surface molecules may affect the immune system response (van Riet et al., 2014).

Both *Streptococcus pyogenes* and *Serratia marcescens* produce exotoxins. *S. pyogenes* produces the pyrogenic exotoxins SpeA, SpeB, and SpeC which have the capacity to unspecifically stimulate CD4+ lymphocytes, leading to a strong secretion of different cytokines (Babbar, 2015). *S. marcescens*, produces prodigiosin, a low-molecular weight red pigmented heterocyclic tripyrrolic toxin with anti-tumor activity (Elahian et al., 2013). The toxins, together with other components of the formulation, result in generation of fever and potential anti-tumor response. The administration route may also influence the efficacy of Coley’s toxins including i.v., i.p., direct injection in the tumor, or subcutaneous or intramuscular administration (Nauts et al., 1946).

A chemical description of “Coley’s toxins” can be assessed using the analytical tools currently used for proteomic and metabolomic studies (Wishar, 2016). Nuclear magnetic resonance (NMR) and mass spectrometry (MS) methods for the analysis of high- and low-molecular weight components of complex mixtures or their derivatives (Alonso et al., 2015) could be used in combination with multivariate analysis to identify the components responsible for anti-tumor activity. The identification of the active components and their mode of action, would allow the selection of more active and better-defined vaccines, as well as the design of tailored formulations capable of producing the right amount of systemic or tumor-localized fever (Noe, 2016) for optimal stimulation of the host immune system and cytokine secretion to achieve best anti-tumor efficacy.

## Author Contributions

MGK received the invitation to contribute in this special issue, designed and wrote most of the sections and supervised the artwork. MM made all the drawings, acquired and analyzed the data and participated in the text writing. FAF wrote the final section and was essential in motivating the team work. RMH inspired the main ideas of this perspective article, supplied relevant literature and critically revised the manuscript.

## Conflict of Interest Statement

The authors declare that the research was conducted in the absence of any commercial or financial relationships that could be construed as a potential conflict of interest.
